# Laughter as a Subject and a Tool for Interdisciplinary Investigations in Philosophy and Neuroscience

**DOI:** 10.1111/ejn.70242

**Published:** 2025-08-25

**Authors:** Vivian Tiemi Sugano, Adriano da Silva Costa, Marilia Biscaia Rizzo, Claudinei Eduardo Biazoli Junior

**Affiliations:** ^1^ Center for Mathematics, Computing and Cognition UFABC Brazil; ^2^ National Institute of Science and Technology on Social and Affective Neuroscience, INCT‐SANI São Paulo Brazil; ^3^ IDOR Pioneer Science Initiative Rio de Janeiro Brazil; ^4^ Hospital Israelita Albert Einstein São Paulo Brazil

**Keywords:** Bergson, cognitive flexibility, continental philosophy, curiosity, laughter, phenomenology

## Abstract

Laughter has been extensively studied by philosophers and neuroscientists, but the potential bridges between these two fields of inquiry have been underexplored. Here, we propose a convergent investigation of the philosophy of laughter and humor, leveraging recent theoretical and methodological advances in human functional neuroimaging. We develop testable hypotheses about the relationships between laughter, global embodied cognitive states, cognitive flexibility, and brain metastability. We argue that laughter, as an eminently embodied set of phenomena, should be better studied using emerging antilocalizationist approaches in neuroimaging, but in a way that integrates phenomenology and the classic findings of localizationist neuroscience. Finally, paralleling the interdisciplinary investigation of curiosity, we argue that laughter with humor is not only a topic but also a tool for advancing joint efforts in neuroscience and philosophy.

## Graphical abstract

Laughter has been explored by philosophers and neuroscientists. Combining the humor theories (incongruity and relief) with neuroimaging advances, we propose that laughter influences brain dynamics and global cognitive states. Laughter is not only a subject but also a tool for advancing neuroscience.

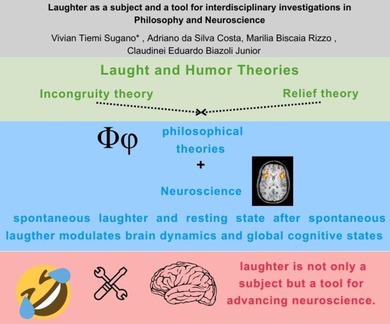

**Abbreviations**
BOLD: blood‐oxygenation‐level‐dependent
DMN: default mode network
fMRI: functional magnetic resonance imaging
fNIRS: functional near‐infrared spectroscopy

## 1 Introduction

Laughter is a set of complex phenomena with a myriad of causes, multiple functions, and effects. These multifaceted acts have long attracted the interest of philosophers (Morreall 1987) such as Aristotle, Kant, Schopenhauer, Nietzsche, Darwin, Freud, Bergson, and Critchley. A recently rediscovered mark on the philosophy of humor and laughter, the work “*Le Rire*” of Henri Bergson (1900) was one of the most influential essays on laughter in the twentieth century. In this essay, Bergson emphasizes laughter as an eminently human and social phenomenon. Contemporary accounts on the philosophy of laughter have revisited and expanded these previous accounts (Critchley 2004).

Laughter has also been a subject of inquiry of experimental psychology, comparative ethology, neurology, and neuroscience. Empirical researchers have proposed that different neural regions and processes underlie the perception and production of laughter. In particular, laughter has been classified as voluntary, also known as non‐Duchenne laughter (e.g., laughter intentionally used in conversation to promote social bonding), and involuntary (or Duchenne) laughter, such as that elicited by tickling (Wattendorf et al. 2019) or the episodes triggered by the humorous content of a joke (Hurley et al. 2011). Far from being a simple response to humor, involuntary laughter is structured and serves specific functions in conversational dynamics (Holt 2012).

Involuntary instances of laughter can indeed be easily perceptually differentiated from voluntary laughter based on acoustic and expression characteristics (Lavan et al. 2015). Involuntary laughter has greater tonal variability, lower average intensity, and greater perceived authenticity, while voluntary laughter tends to be more nasal and less emotionally engaging. Differences in production directly influence emotional perception and authenticity judgments, with the physiological markers of spontaneous laughter more closely linked to genuine emotional responses (Lavan et al. 2015).

Converging with Bergson's philosophy, current research on the affective and social neuroscience of laughter in humans (Provine 2001; Davila‐Ross and Palagi 2022; Nummenmaa et al. 2023) also recognizes laughter as an eminently social behavior embedded in social and linguistic contexts (Provine 2004, 2016; Scott et al. 2014, 2022). Notably, human laughter can be dissociated from specific affective phenomena, particularly from humor. For instance, individuals' intentions when laughing may diverge from the apparent emotional context; then, laughter can be used for intentional deception purposes (Bryant and Bainbridge 2022). Laughter is indeed deeply embedded in specific social frameworks, requiring reciprocity, a characteristic sharply in contrast with homologous vocal behaviors in nonhuman primates (Curry and Dunbar 2013; Nummenmaa et al. 2023).

The emerging field of *human* affective and social neuroscience, which uses mainly noninvasive functional neuroimaging techniques, has revealed some core aspects of laughter complexity (Davila‐Ross and Palagi 2022; Nummenmaa et al. 2023). However, most neuroimaging studies focus on mapping individual brain correlations of laughter perception (Nummenmaa et al. 2023), mainly due to the extremely high sensitivity of functional magnetic resonance imaging (fMRI) to movement artefacts. In fact, movement artefacts preclude investigating laughter production using fMRI, though alternative functional neuroimaging techniques based in the same neuro‐hemodynamic coupling phenomena (i.e., functional near infrared spectroscopy, fNIRS) would in principle allow probing brain function during laughter production (Balardin et al. 2017). Importantly, fNIRS also allows recording brain function in freely moving participants and is easily adapted to simultaneous neuroimaging of dyads (i.e., hyperscanning—Bazán and Amaro 2022).

Though not yet properly applied to the human neuroimaging of laughter, recent theoretical and experimental advances in fMRI and fNIRS allow for antilocalizationist and postreductionist investigations of complex behaviors and phenomena (Pessoa 2022; Gomez‐Marin 2022; Noble et al. 2024). In light of these theoretical advances, mainly based on dynamical systems modelling of whole‐brain dynamics and the emerging possibility of probing brain function in real‐world settings with less susceptibility to motion, we argue for a renewed philosophically informed neuroscientific investigation of laughter in human neuroimaging. Here, we exemplify this new venue of investigation by putting forward hypotheses on laughter production and effects and outlining an illustrative experiment designed to test these hypotheses.

For this, we first present some remarks on contemporary philosophical and theoretical accounts on laughter, followed by a brief overview of the current evidence for the neural bases of laughter production. Then, an embodied cognition and dynamical systems take on laughter production is used to elaborate some specific predictions on the global brain functional dynamics during and after spontaneous laughter. Finally, we argue for promoting laughter not only as a privileged subject (Provine 2016) but also as a tool for interdisciplinary investigations in philosophy and neuroscience, using recent efforts on the neuroscience and philosophy of curiosity as a blueprint (Zurn and Bassett 2023).

## 2 A Few Remarks on the Philosophy of Laughter

In the seminal work of Morreall (1987), three theories of laughter were proposed: the Superiority Theory, the Relief Theory, and the Incongruity Theory. In the Superiority Theory, represented in the works of Plato, Aristotle, and Hobbes, laughter arises from a sense of triumph or superiority over others or over our past selves. In the Relief Theory, laughter is thought of as a physical release of accumulated psychological energy, with Spencer and Freud as the main proponents. Finally, the Incongruity Theory asserts that laughter emerges from the perception of something that deviates from our expectations. This theory is associated with philosophers like Pascal, Kant, and Schopenhauer.

In fact, these traditional theoretical lines are not mutually exclusive; hybrid theories can be observed in authors such as Descartes and Bergson. The three classical theories can actually be applied to different aspects or instances of laughter. In line with both the Incongruity and Relief Theories, for instance, tickle‐induced laughter is triggered by sensory incongruities and leads to experienced relief afterward (Wattendorf et al. 2013, 2016, 2019).

Recently rediscovered, Bergson's works represent a hybrid theory of laughter. In line with the Superiority Theory, Bergson proposes that laughter corrects *inferior* behaviors marked by rigidity. Bergson also proposes that we humans laugh at what is human or what reminds us of the human, such as a dog dancing on two legs. Throughout his *Le Rire* essay, Bergson points to laughter as a way of reducing social tensions, in line with the Relief Theory. Both in funny everyday scenes and in comic texts in plays, laughter brings a malleability to the rigidity of the social fabric. We laugh, for example, of the mechanical repetition of movements and words: “The comic is this side of the body which resembles a thing, this aspect of events which imitates, by its rigidity of a very particular kind, pure and simple mechanism, automatism, lifeless movement, in short. It therefore expresses an *individual or collective imperfection* that calls for immediate correction. Laughter is precisely that correction. Laughter is a social gesture …” (Bergson 2018, 75).

Expansions and adaptations of these traditional philosophical theories in fields such as linguistics (Attardo 1994), anthropology (Plessner 1970), psychology (Mcgraw and Warner 2014), and comparative ethology (Palagi et al. 2022) have been developed. In linguistics, the Incongruity Theory served as a base for the development of the widely accepted Script‐Based Semantic Theory of Humour (SSTH), proposed by Raskin (1985), that was further expanded in the General Theory of Verbal Humour (Attardo 1994). A theoretical development in psychology, the Benign Violation Theory suggests that laughter occurs when a norm is violated in a way that is simultaneously nonthreatening (Mcgraw and Warner 2014). In ethology, Palagi et al. (2022) propose that laughter is a multifaceted evolved social behavior that hence occurs in other species and is not necessarily related to semantic context. The function of laughter for Palagi et al. (2022) is to regulate social relationships, reduce tensions, and strengthen social bonds in humans and other social animals.

Plessner (1970) proposes a phenomenology of the body in laughing based on the idea of an eccentric positionality of the human being, who *is* a body and *has* a body at the same time. Hence, the human being is both a subject and an object of experience. When the balance of this dual‐awareness of being and having a body is disrupted, suddenly revealing our singular mode of embodiment, laughter erupts. This imbalance can arise from incongruity or social tension, for instance, and leads to a temporal loss of control that further reveals the body's autonomy. Laughter cannot be reduced to either a rational or emotional response (Plessner 1970, 65): “Response to the comic comes from the whole man”. Plessner also argues that laughter connects us to the world: “The laughing person is open to the world. The consciousness of separation and isolation which frequently appears as a consciousness of superiority signifies at one and the same time detachment from the given situation and *openness or flexibility*. Disengaged in this way, man tries to engage others. And it is not a matter of chance that the outbreak of laughter begins immediately, more or less”apoplectically,“ and, as if to express the openness of the laugher, rings out into the world as he exhales.”

Simon Critchley, a contemporary English philosopher, argues that there is a kind of social agreement within a group when a joke is told: “(…) for the incongruity of the joke to be seen as such, there must be a congruity between the structure of the joke and the social structure ‐ without social congruity, there is no comic incongruity” (Critchley 2004, 4). He also explains that in order to understand laughter, we need to study it socially; there must be common ground in people's lives for there to be collective laughter.

Critchley (2004) quotes the philosopher‐comedian Eddie Waters: “(…) But a real joke, a comedian's joke has *to do more than release tension*, it has to release will and desire, it has to change the situation” (p. 3). Critchley then proposed that laughter can change situations and the way we perceive and interact with the world. Therefore, for Critchley as well as for Bergson, laughter is an important social *tool*, both for modifying and reinforcing worldviews.

## 3 A Brief Overview of the Neural Bases of Laughter Perception

Despite the embodied, embedded, and prominent social character of laughter, conventional neuroscience is epistemologically committed to describing human acts in isolated individuals and with a focus on localizing brain regions correlated with isolated aspects of phenomena. However, localizationist neuroscience findings in isolated individuals can provide invaluable insight for advancing new interdisciplinary investigations of laughter. In this section, we focus on the evidence for the neural bases of the individual act of laughing in humans, that is, the neural mechanisms underlying laughter production.

Though little direct evidence on the neural bases of laughter production has been provided by noninvasive functional neuroimaging studies due to technical limitations, other lines of investigation have greatly advanced our understanding of the functional neuroanatomy of laughter. Some of these studies have also provided important contributions to the phenomenology of laughter and humor by presenting first‐person accounts of direct brain stimulation experiences, for instance.

Clinical observations and electrical stimulation studies have particularly contributed to this emerging understanding of the neural bases and phenomenology of laughter. Individuals with Parkinson's disease treated with deep brain stimulation of the subthalamic nucleus can be induced to laugh by direct stimulation (Krack et al. 2001). Importantly, unlike instances of pathological laughter, participants reported feelings of joy. In a revision of human intracranial electrophysiology results, Guillory and Bujarski (2014) identified two distinct types of laughter induced by direct brain stimulation: laughter with mirth and laughter without mirth. Laughter with mirth, characterized by the accompanying emotional experience of joy, is elicited by stimulations of the left inferior temporal lobe, nucleus accumbens, anterior limb of the internal capsule, subthalamic nucleus, fusiform gyrus, parahippocampal gyrus, and left inferior frontal gyrus. In contrast, laughter without mirth is elicited by stimulating the supplementary motor area and premotor cortex.

In some patients with epilepsy, electrical stimulation of the pregenual anterior cingulate cortex elicited laughter (Caruana et al. 2016), with half of them reporting that the laughter was accompanied by a sense of joy. Electrical stimulation in other areas including the anterior cingulate cortex, frontal operculum, and temporal pole also elicited laughter or smiling (Caruana et al. 2020). The finding of induced laughter by direct stimulation of a specific region of the anterior cingulate cortex has been recently replicated (Zauli et al. 2022). Individuals described experiencing an uncontrollable and inexplicable urge to laugh, accompanied by a sensation of joy (Zauli et al. 2022).

The studies by Zauli et al. (2022), Caruana et al. (2015, 2020), and Krack et al. (2001) underscore the importance of considering the first‐person accounts on the study of laughter. These first‐person accounts of emotional experiences accompanying the physical act of laughter induced by the direct stimulation of a specific brain region provide strong evidence for a specific neural circuitry for involuntary laughter production (Zauli et al. 2022). However, we would argue that these specific findings can also be viewed from large‐scale brain networks, whole brain, or embodied dynamics perspectives (Pessoa 2022).

Combining previous brain lesions evidence with resting‐state fMRI modeling using a network–symptom–lesion mapping approach, Klingbeil et al. (2021) investigated the neural bases of pathological laughter and crying. A bilateral network with positive resting‐state functional connectivity between cingulate and temporomesial cortices, striatum, hypothalamus, mesencephalon, and pons, and negative connectivity to the primary motor and sensory cortices was identified.

Gerbella et al. (2021) conducted a multifiber tractography study in healthy individuals and proposed two networks associated with laughter. The first would be linked to involuntary laughter and involves the pregenual anterior cingulate cortex, ventral temporal pole, and the nucleus accumbens (part of the ventral striatum). The second would be related to voluntary laughter and includes the frontal operculum and primary motor cortex. These networks would interact through the presupplementary motor area, which connects to both the anterior cingulate cortex and frontal operculum. As an independent evidence for the role of the nucleus accumbens in emotional laughter, Manninen et al. (2017) found that social laughter increases endogenous opioid release in brain regions associated with reward processing using positron emission tomography. This study also found that baseline availability of Mu‐opioid receptors in the orbitofrontal cortex and cingulate cortex predicts social laughter frequency: individuals with higher receptor availability were more likely to laugh in social contexts.

Despite the limitation imposed by the marked sensitivity of fMRI to motion, some functional neuroimaging studies on laughter perception have been conducted. Meletti et al. (2015) used fMRI to study emotion‐induced cataplexy in children, observing increased BOLD activity in a network comprised of the amygdala, ventromedial prefrontal cortex, anterior insular cortex, and nucleus accumbens. When laughter was not followed by cataplexy, bilateral motor and premotor areas and the anterior cingulate gyrus were recruited instead. Talami et al. (2020) investigated neural correlates of involuntary laughter across different age groups using fMRI. Activation of the motor network, anterior cingulate cortex, amygdala, nucleus accumbens, hippocampus, basal ganglia, and thalamus, as well as the cerebellum, was observed. Age‐related differences were also observed: Younger participants showed greater involvement of the reward circuit (nucleus accumbens), whereas older participants exhibited stronger activation of the default mode network (DMN).

As highlighted in this brief review of studies on the neuroscience of laughter perception, most investigations have been conducted within a localizationist framework (Noble et al. 2024), aiming to map specific brain regions associated with laughter perception and to dissect circuits instantiating voluntary and involuntary laughing. Alternatively, laughter can be investigated from an embodied perspective associated with methods to reveal emergent patterns of global brain dynamics rather than localized activity.

## 4 New Approaches to the Human Neuroscience of Laughter: Embodied Cognition and Dynamical Systems

Laughter is essentially a bodily matter (Critchley 2004). As an embodied phenomenon and in contrast to smiling, laughing is similar to crying and to orgasm: convulsive phenomena associated with a transient loss in self‐control (Plessner 1970). In Critchley words, this class of phenomena that includes laughter can be described as an “explosion expressed with the body” (Critchley 2004, 9).

Embodied accounts have been recently approximated to current theorizing and practice in human functional neuroimaging. Käufer and Chemero (2021) have indeed argued that embodied cognition theories are contemporary expressions of the phenomenological tradition in cognitive science and neuroscience. According to them: “The success of this future phenomenology depends upon its ability to show how higher‐order experiences emerge from naturalistically conceived self‐organizing systems. This, in turn, depends on the willingness of cognitive scientists to take phenomenology seriously and their willingness to collaborate with philosophers” (Käufer and Chemero 2021). Methodologically, integrating careful phenomenological descriptions with dynamical systems modelling have been proposed as the most suitable way to close the gap between philosophical and neuroscience investigation of embodied and situated behaviors (Käufer and Chemero 2021).

Much of the human neuroimaging literature on laughter focuses on mapping or localizing brain regions correlated with laughter perception (Nummenmaa et al. 2023). Importantly, as movement artefacts are an intrinsic issue of most commonly used neuroimaging techniques, direct measures of brain function during laughter production are virtually not feasible in fMRI research. Moreover, classical human neuroimaging investigations of laughter presuppose clearly distinct and dedicated neural circuits (Provine 2001). Phenomenological investigations, on the other hand, emphasize its dynamical, individual, and interactive nature (Prusak 2006).

In the last couple of decades, the focus of the neuroimaging community has indeed changed from localizing brain function to understanding functional dynamics. Accordingly, methods of acquisition of functional neuroimaging now more commonly use “resting‐state” or “naturalistic” stimuli (i.e., movies) rather than asking participants to perform specific cognitive tasks. At rest, brain dynamics is “more random” than during the performance of a specific task (Capouskova et al. 2023). This increased flexibility of brain dynamics in less constrained settings has been associated with greater cognitive flexibility, leading to the learning of new tasks, though with less precision and specialization (Capouskova et al. 2023). This cognitive and brain functional state of increased flexibility is expected in settings where no immediate and specific response is required, as in resting‐state or watching a movie (Capouskova et al. 2023). More precisely and reliably, the brain dynamics variations can be better captured by the concept of metastability (Hancock et al. 2025).

## 5 Laughter as a Subject: Philosophically Based Brain Dynamics Hypotheses

Focusing on large‐scale networks or whole brain dynamics rather than on localizing brain function is a feasible and meaningful way to integrate phenomenology with neuroimaging in the investigation of laughter production. For this, dynamic systems derived metrics have been proposed as the most suitable way to characterize the relation between brain dynamical activity and complex behaviors (Tognoli and Kelso 2014).

Metastability is a concept derived from dynamical systems theory and represents a metric for the balance between integration and segregation in whole‐brain and large‐scale neural networks (Hancock et al. 2025). Metastability is an emergent property of the collective behavior of neural populations. In whole‐brain computational models based on individual structural connectivity neuroimages, metastability encodes the level of exploration of the repertoire of dynamic states constrained by the brain's structural connectome (Deco and Kringelbach 2016; Kringelbach and Berridge 2017).

Mounting evidence shows that proxy measures of brain metastability in functional neuroimaging are associated with specific global cognitive states, reliably and consistently varying across conditions such as specific cognitive tasks (Hellyer et al. 2015), sleeping (Cavanna et al. 2018), meditating (Escrichs et al. 2019), watching movies (Kringelbach et al. 2023), under the action of psilocybin (Carhart‐Harris et al. 2014), and simply resting (Capouskova et al. 2023; Escrichs et al. 2019; Hellyer et al. 2015; Kringelbach et al. 2023). During tasks requiring focused attention, metastability is consistently lower than during the resting state. Recently, Kringelbach et al. (2023) found that metastability is significantly higher during film watching than during rest.

Although a clear picture of the role of brain metastability in cognition is still emerging, both computational modeling studies based on structural MRI and fMRI evidence associated with different experimental conditions suggest that metastability encodes *global cognitive states and the flexible switching between such global states* (Hellyer et al. 2015). Specifically, metastability has been proposed to instantiate two fundamentally different cognitive modes: (Attardo 1994) An online, reduced metastability mode, caused by activation of attentional networks and deactivation of the DMN, in which a momentary embodied coupling with the immediate environment is achieved, and which is associated with the successful completion of a given task; and (Balardin et al. 2017) an offline, increased metastability mode, caused by activation of the DMN, characterized by a decoupling from immediate environmental affordances, allowing for reflection, detached description, and planning. These proposed two global cognitive modes are rooted in the Heideggerian distinction between two ways of being, with *Zuhanden* representing an embodied and engaged mode and *Vorhanden* a disengaged relation to the affordances (Käufer and Chemero 2021). This distinction between two modes of being can also be approximated to the idea of the eccentric positionality (the body taken as an object and an embodied subject) of human beings and its role in laughter (Plessner 1970).

Accordingly, higher metastability has been proposed to instantiate cognitive flexibility and to be associated with “relaxed” and detached cognitive states. Cognitive flexibility is an executive function that is closely linked to creativity (Diamond 2013). According to Diamond, the terms cognitive flexibility and creativity, task switching, and set shifting overlap and intertwine, stating that cognitive flexibility is the opposite of cognitive rigidity.

If brain metastability instantiates cognitive flexibility and the switch between online and offline global cognitive state, this metric can be used to empirically test, using neuroimaging data, at least some predictions of the theories on laughter. We therefore have advanced some philosophically based hypotheses for the relation between laughter and brain metastability: (Attardo 1994) During the production of involuntary laughter, metastability will be greater than in resting‐state due to the predicted increased flexibility (proposed by the Incongruence Theory and Bergson's proposal); and (Balardin et al. 2017), after laughing, if Relief Theory is correct, metastability should increase even further. This second prediction is in line with recent results of increased metastability in “relaxing” movie‐watching when compared to rest (Kringelbach et al. 2023) and on previous theoretical work proposing increased metastability as part of a causal mechanism of eudaimonia (Kringelbach and Berridge 2017).

Importantly, these predictions are pragmatically testable in less constrained settings using fNIRS. In contrast to fMRI, functional near‐infrared spectroscopy (fNIRS) offers distinct advantages for studying laughter in more ecologically valid contexts. fNIRS is based on the same neurovascular mechanisms as fMRI but is less susceptible to motion artifacts, making it particularly well‐suited to studying laughter (Balardin et al. 2017; Sakai 2022). We have detailed such an fNIRS experiment in a registered report (Sugano et al. 2024). Shortly, a rest–task–rest experimental paradigm would allow the investigation of laughter effects on global brain dynamics by comparing metastability before laughing (baseline), during spontaneous laughing production (triggered by humorous videos) and after laughter, characterizing the full cycle of pleasure (Kringelbach and Berridge 2017). Moreover, control conditions for voluntary laughter and watching nonhumorous videos would allow testing whether possible observed differences are due to movement itself or other stimulus features.

However, fNIRS recordings are limited to sparsely sampled cortical areas. Then, further investigations on the dynamics of subcortical networks or of the potential mechanistic integration between activity in specific networks instantiating laughter and global brain dynamics would require simultaneous stimulation and neuroimaging acquisitions. fMRI studies and computational modeling could also be applied to indirectly test a hypothesis in which the involuntary laughter network modulates DMN regions that in turn determine global brain metastability (Langdon et al. 2023). In fact, a theoretical framework for the potential mechanisms integrating mesocorticolimbic networks that instantiate pleasure cycles and global metastability has already been proposed (Kringelbach and Berridge 2017).

Importantly, we propose that careful first‐person description of the experiences during data acquisition are crucial to investigate both the relation between laugh and concurrent affective states and the phenomenology of global cognitive states. As proposed by Hurley et al. 2011 (p. 20): “To determine whether there really was humour in the things that subjects laughed at, a researcher would need to interview the people who laughed and ask, one way or another, if they felt that something was funny when they laughed; and, if so, what was funny, and why? (It might not be at all obvious to the researcher, but very obvious to the in‐group being studied).”

Even more importantly, new empirical approaches properly addressing the central social nature of laughing are warranted, for which fNIRS hyperscanning and the study of between‐brain emerging properties are desirable and feasible extensions (Bazán and Amaro 2022; Chidichimo et al. 2024).

## 6 Laughter and Humor as Tools for Integrating Neuroscience and Philosophy: Interdisciplinarity, Curiosity, and Partnership

Curiosity is the intrinsic drive that makes us move in a self‐directed and purposeful way and leads to a process of building networks of growing knowledge for the mental construction of the world (Patankar et al. 2023). Both the attempt to fill the gaps in understanding—information gap theory (Patankar et al. 2023) and to discover the latent organization of the world—compression progress theory (Patankar et al. 2023), become impulses of curiosity that build expansive, complex, and flexible knowledge networks (Patankar et al. 2023). Zurn and Bassett (2023) argue that the very concept of possibility is intertwined with the notion of curiosity, since both work together, and curiosity prevents possibility from going unnoticed by us. Curiosity of the critical kind brings the possibility of investigating and questioning the present and exploring the world in its most diverse forms in an attempt to understand how it could be constructed or understood (Zurn and Bassett 2023). Curiosity, by allowing experimenting with new conceptual formulations, breaks with old social customs and normative structures, enabling real advances in different spheres, including science (Zurn and Bassett 2023).

This possibility of proper innovation in knowledge, stemming from curiosity, is aligned with one of the functions of humorous laughter. In both cases, there is an attempt to correct a rigidity that limits our understanding, creating possibilities for creativity. Theories of humor are sometimes called theories of laughter, as they seek to understand what makes humans laugh. When emitted spontaneously, laughter is often contagious and creates affiliations within groups (Provine 2001). Therefore, humor can reveal congruences through group laughter (Mcgraw and Warner 2014). Laughter can also reveal incongruences and bring epistemic tensions to light in a lighthearted way (Critchley 2004). According to Critchley (2004), a true joke must do more than just lighten a situation; it must also open the possibility of changing it. When humor exposes an incongruence and we laugh, the underlying assumption of congruence of a given group is brought to the surface.

As previously proposed in an interdisciplinary investigation on the neuroscience and philosophy of curiosity (Zurn and Bassett 2023), we propose that humorous laughter can be an important tool to theorize. The spontaneous humorous laughter is possibly related to increasing cognitive flexibility, an executive function closely linked to creativity. The hypothesis that metastability increases when we laugh spontaneously has yet to be tested, laughed at if wrong, and published as a negative result if so to avoid others in pursuing deadens.

Indeed, dialogical interaction between groups and individuals from different backgrounds, such as neuroscientists and philosophers, can often be tense and marked by misunderstandings on both sides. This tension can hinder the exchange that should occur in interdisciplinary efforts and thus limit the expected advancement of knowledge. Laughing together, and particularly laughing at ourselves, recognizing the incongruity revealed by humor, can be a crucial tool in overcoming this problem. Bergson, as previously presented, argues that laughter brings malleability to the social fabric, and laughing together can make the environment “lighter.” Critchley (2004) argues that a joke can burst the “bubble of tension,” causing those who hear it to experience a pleasurable effect, a “comic relief.”

Laughter can connect people, it is like a game where you throw the ball and the other person catches it and throws it back (Critchley 2004). Critchley (2004) quotes anthropologist Mary Douglas (p. 10): “A joke is a play upon form that affords an opportunity for realizing that an accepted pattern has no necessity”. In other words, a joke can ridicule patterns and rituals that we have created socially and raise questions about our own attitudes. It can also change worldviews. If we laugh together, legitimately, at a joke that questions the *sensu comunis*, we are together subtly agreeing with the joke. Laughing then can unite people and at the same time question the underlying assumptions of diverse groups, potentially opening up spaces for interdisciplinarity.

## 7 Conclusions

The bridge between neuroscience and philosophy is essential, albeit tense, and if this bridge could be built with some humor and laughter, the path will be richer, more cohesive, creative, and innovative. Here, we illustrate a possible bridge between neuroscience and the philosophy of laughter and humor by advancing hypotheses about how laughter modulates flexibility and the brain dynamic marker of metastability. We also suggest that laughter can be not only a privileged subject but also a crucial tool for interdisciplinary inquiry in philosophy and neuroscience, particularly in the emerging convergence of phenomenology, embodied cognition, dynamical systems modelling, and human functional neuroimaging.

## Author Contributions

**Vivian Tiemi Sugano:** conceptualization, investigation, methodology, writing – original draft. **Adriano da Silva Costa:** formal analysis, validation, writing – review and editing. **Marilia Biscaia Rizzo:** conceptualization, investigation, methodology, writing – original draft. **Claudinei Eduardo Biazoli Junior:** conceptualization, methodology, supervision, writing – original draft.

## Conflicts of Interest

The authors declare no conflicts of interest.

## Peer Review

The peer review history for this article is available at https://www.webofscience.com/api/gateway/wos/peer‐review/10.1111/ejn.70242.

## Data Availability

Data sharing is not applicable to this article as no new data were created or analyzed in this study.
